# Twenty-First Annual Meeting of Presidents

**Published:** 1921-01-01

**Authors:** 


### THE LEAGUE OF MERCY.

### Twenty-first Annual Meeting of Presidents.

The twenty-first annual meeting of Presidents of the League of Mercy was held at St. James's Palace on Monday, December 20, and was presided over, in the absence of H.R.H. the Prince of Wales, by the Lady Grand President, H.R.H. Princess Alice, Countess of Athlone. H.R.H. was supported by H.H. Princess Helena Victoria and H.H. Princess Marie Louise, while among those present were the Dowager Lady Dimsdale, Sir William and Lady Collins, Lord Farquhar, Lady Alexander, and Lady Tree.

The minutes of the last meeting having been read and approved,

Mr. Montagu Sharpe, K.C., D.L., moved: "That the Rt. Hon. the Viscount Farquhar, G.C.V.O., be appointed Chairman, Sir Fredk. Green, K.B.E., Deputy-Chairman, and Mr. Arthur H. Greening, Hon. Secretary, respectively." He said Lord Farquhar had been associated with the League eighteen years, and had come to be regarded as one of its principal pillars, and workers in the League did not forget that the doors of his house were always open for meetings to further its objects.

Mrs. Gerald Buxton seconded, and it was carried.

Lord Muir Mackenzie, G.C.B., proposed: "That H.H. Princess Marie Louise, Colonel the Rt. Hon. Lord Lamborne, the Dowager Lady Dimsdale, O.B.E., Viscount Hill, Lady George Lawson-Johnston, and the Rev. H. Hornby-Steer, M.A., be appointed members of the Executive Council." He said there was no need to descant on the qualifications and the merits of those whose names were included in the resolution, though to do them justice would occupy some time. All present could feel that the affairs of the League, which in the past had been so ably conducted, would be equally well looked after in the future—i.e., during a time when the need would be greater than had yet been experienced. He could almost wish that the Executive Committee were really executioners, so that they might execute condign punishment upon those rich people who could be seen to be flaunting almost every extravagance before one's eyes, except the effort at providing adequately for the sick and needy.

Colonel Sir Charles Wakefield, Bart., C.B.E., seconded, remarking how grateful the League felt to these ladies and gentlemen for having placed their consecrated imaginations at the disposal of the League.

The resolution was carried.

Presentation of the Bar of the Order of Mercy.

H.R.H. the Grand President then presented the Bar of the Order of Mercy to the following ladies and gentlemen:—

*Lady Presidents.*—The Countess Grosvenor, St. George's, Hanover Square; the Hon. Lady Lawson Johnston, East St. Marylebone.

*Vice-President.*—The Rev. C. J. Smith, Hammersmith.

*Lady Vice-Presidents.*—Lady Clayton, Buckinghamshire; Miss Cayley, Fulham; Mrs. J. D. Charrington, Kingston-Surbiton with Wandsworth-Putney; Mrs. Harold Hardy, West St. Marylebone; Mrs. Horan, Tonbridge; Mrs. Ray Linsham, Deptford-Brockley.

*Members.*—Miss Selina Pyke, West St. Pancras; Miss Ellen A. Symons, Eastbourne.

The Order of the League was presented to the following:—

*Lady Presidents.*—Lady Gibbons, Chertsey-Woking; Lady Tree, Lewisham; Mrs Cain, Hitchin.

*Vice-Presidents.*—Sir John Wood, Bart., Stowmarket; Dr. Hewkley, St. Marylebone East; Mr. Fowler Pettie, Battersea.

*Lady Vice-Presidents.*—The Dowager Lady Greenwell, Epsom-Esher; Mrs. Walter Carlile, Buckinghamshire; Mrs. A. G. Cocke, St. Pancras North; Mrs. Drummond, Brighton-Hove; Mrs. L. Frank, South Paddington; Mrs. E. C. Harrison, Fulham.

*Members.*—Mr. A. E. Core, Strand; Mr. F. Overton King, Islington North and West; Miss Adams, Bedford and Mid Beds; Mrs. Vincent Baxendale, St. George's, Hanover Square; Miss Blissett, St. Marylebone East; Miss Buxton, Clapham; Miss Chalmers, Romford; Miss A. Chaplin, Epsom-Esher; Miss Monica Cosens, Lewisham; Miss L. Cowie, Brentford; Mrs. Edith Cox, Dulwich-Norwood; Mrs. Duke, Eastbourne; Mrs. Everitt, St. Marylebone East; Mrs. C. Ewart, St. George's, Hanover Square; Mrs. R. Garrett, Horsham; Mrs. S. W. Godin, Kingston-Surbiton; Miss H. H. Hamilton, Kensington South; Miss Hornby, Lewisham; Mrs. Howard, Epping; Mrs. P. Hyman, Kensington South; Miss H. Lucas, Kensington North; Miss Mitchell-Innes, St. Marylebone West; Miss M. Montgomery, Chelsea; Mrs. Gordon Mosley, Hampstead; Miss Ravenshaw, East Grinstead and Haywards Heath; Miss E. J. Reeve, Epping; Mrs. Stickings, Westminster; Miss Strapp, Kingston-Surbiton; Mrs. Edwards, Fulham; Miss Sophie Turner, Croydon; Miss Davis, Paddington South.

Address by the Lady Grand President.

H.R.H. Princess Alice, Countess of Athlone, said: My lords, ladies and gentlemen, I am here to-day as a substitute—and many of you must think a very poor substitute (No, no)—for the Prince of Wales; but I am the bearer to you of every message of hope and confidence and congratulation from him. Before saying one word of my own you will let me read to you a letter from the King:

Buckingham Palace,
December 20, 1920.

The Queen and I are pleased to learn that, notwithstanding many adverse circumstances during the last twelve months, the League of Mercy continues successfully to maintain its work in support of the voluntary hospitals, and that during the twenty-one years which have elapsed since King Edward founded the League more than £300,000 has been collected through its organisation and distributed to the London hospitals, while more than £37,000 has been distributed among hospitals outside London.

I cordially congratulate all those who have worked for the League on the success of their labours.

(Signed) George R.I.

The most interesting facts to be recorded at meetings are apt to lie in the financial situation. I will not anticipate in any way the statement which our inestimable Treasurer, Sir Frederick Green, will presently submit to you, but I dare to think that all who are in any way in touch with his work will hear it with sincere satisfaction.

The Garden Party held, by permission of the King, in St. James's Palace last July, was a marked and remarkable success; it enabled the old and constant friends to knit anew their ties of friendship and interest; it stirred us all to fresh efforts, which have already proved fruitful, and it brought us all into line, a line of advance, in our great and good cause, which we hope will suffer no check. Indeed, the only blot on that happy occasion was that, once more, the irreplaceable Prince of Wales was obliged to be represented by myself. (Laughter.)

It is quite unnecessary for me to rehearse to you the Charter, or to recall to you the Constitution of the League. Its direct and noble aims are well and widely known; but I must repeat to you its primary need of money. And in this respect will you forgive me if I underline the spade-work, which is most necessary and really most beneficial.

Presidents and Vice-Presidents use their own discretion as to the methods and organisation most suitable to their several districts, and have in many instances raised considerable sums by entertainments, in addition to collections by members of small contributions from Associations in the manner contemplated by the Statutes of the League.

We have reason to be very grateful to those who have

**The League of Mercy**—(*continued*).

promoted and patronised functions and entertainments, but our deeper debt of gratitude will always lie with those who have carried our message, so to speak, from house to house, who have burnt into the minds of people in every class and phase of life how they can, according to their most modest circumstances, give a real impetus to our work, the work of supporting the voluntary hospitals in London through the King's Fund and other hospitals outside London by direct assistance.

There is a not unreasonable feeling just now that the League of Mercy has received the reverse of preferential treatment from King Edward's Fund for this reason. Applications have been made from time to time for permission to make collections for the League in County Council schools, but they have been refused. Early this year, however, it came to our knowledge that a proposal was being considered by the London County Council and its teachers for making school collections for the hospitals, and that the King Edward's Hospital Fund had been apprised of this proposal. The Council of the League at once took steps to bring to the notice of the L.C.C. facilities which the organisation of this League provided for such purpose, and to remind the King's Fund that collection of small sums from large numbers of persons fell within the province of the League as prescribed by its Charter. The King's Fund replied that they were merely asked to receive such sum as might be sent them for distribution, and were not consulted as to the method of collection.

The receipts of the King's Fund have this year been augmented by £10,348, derived from the school children's collections, while a similar amount was allocated by the schools themselves to particular hospitals. Here again some co-ordination of effort might result in securing larger support for the hospitals without overlapping or conflicting appeals from different organisations or sources, and with due regard to authorised methods of collection. The matter is, of course, quite arguable from two points, but I submit to your notice the large sums collected as a proof of what the smallest and weakest in the community can do for a good cause.

The cardinal danger inherent in all voluntary organisations is, of course, overlapping. No body of persons could, or should, be more forward than the League of Mercy to applaud and appreciate the splendid work of the Red Cross; but even here one remembers that whereas the Charter of 1908 laid down as its primary object to furnish aid to the sick and wounded in time of war, it obtained, in 1919, a new Charter, extending its powers to "the improvement of health, the prevention of disease, and the mitigation of suffering throughout the world," thus incorporating the words of Article 25 of the Covenant of the League of Nations. These wide powers would enable the Red Cross Society, if it were so disposed, to seek to supplant all agencies working for the same humane objects, and to maintain hospitals not only in London and the United Kingdom, but throughout the world.

It will be remembered that the Charter of the League of Mercy, granted in 1899, besides authorising it to promote the welfare of King Edward's Hospital Fund, directed the League in every way, and especially by encouraging personal service on the part of large numbers of persons, to further the interests and promote the adequate maintenance of hospitals and other institutions for the relief of sickness and suffering, especially such as are supported by voluntary contributions. When at the beginning of this year the British Red Cross Society entered upon its peace work under its new Charter, it appears to have been unaware of the collection and distribution for extra-Metropolitan hospitals which the League of Mercy has undertaken for a great many years. A friendly conference was held, and the co-operation of the League in their October appeal was invited by the Red Cross Society, who agreed to share in the results of the joint appeal received by the League of Mercy, amounting to no less than £4,000, and I am glad to say a cheque for that amount has been received to-day. (Applause.)

The Council of the League has been considering a concordat between such voluntary agencies as the British Red Cross Society, the League of Mercy, and other similar funds, to obtain the maximum benefit for the hospitals with quite a negligible amount of friction between the organisations who are really "out" to help them. We hope to come to some definite arrangement in the near future.

We know that while at many voluntary hospitals there are long waiting-lists of patients anxious to be admitted, there are some thousands of vacant beds in Poor-Law hospitals. Was this in King Edward's mind when in establishing his Hospital Fund and League of Mercy he declared, "Public opinion has shown itself upon more than one occasion in favour of the maintenance of the voluntary system"? One thing is certain: the voluntary institutions present far greater attractions than those maintained out of public funds.

And—and this, ladies and gentlemen, is my last word—surely no great gathering of this League should occur without a reverent allusion to its great founder. As one reviews these last years—years crowded with suffering, yet rich in opportunity to relieve it—one realises what the hospitals and those who have come under their healing influences owe to King Edward, to his deep benevolence, his wise forethought. This is a note of gratitude in our work of harmony which must never be allowed to grow dull or dumb; it is a note which has been rightly struck anew by the Prince of Wales in a letter which I will now ask you to allow me to read:

St. James's Palace,
December 16, 1920.

I greatly regret that I am not able to be present at the Annual Meeting of the Presidents of the League of Mercy on this occasion. It was a real pleasure to me as Grand President of the League to take the chair at the twentieth meeting of Presidents last year, and to learn the splendid record of success which has attended the League since it was initiated by my grandfather in 1899. The voluntary hospitals deserve and need all the financial support which such an organisation as the League of Mercy is able to accord them, especially during the period of stress and uncertainty through which they are now passing.

The League of Mercy seems to me to be especially adapted to securing for the hospitals larger financial support from all classes of the community, and it will be a source of gratification to me to learn that its membership is further increased, and that it will in this way contribute to the maintenance of the voluntary hospitals as King Edward so earnestly desired when he inaugurated the Fund which bears his name.

(Signed) EDWARD P.

Sir FREDERICK GREEN then gave a review of the work of the League during the past year. He thought it would be agreed that the results achieved were distinctly satisfactory. The total reached the substantial sum of £25,483. (Applause.) He regretted that only about half of that amount had been actually received, and he urged a more prompt remittance so that the money could be dealt with without delay. He specially mentioned the three districts which occupied top positions by virtue of the sums collected: South Kensington, £1,350; South Paddington, £1,072; Epsom and Esher, £977. In South Kensington Princess Marie Louise had displayed great activity in organising the work of her district; and South Paddington was a centre of energy of the Dowager Lady Dimsdale, a large part of the money having been collected in small amounts. The League felt very grateful to the Mayor of Lewisham who, with Lady Tree's assistance, organised a "League of Mercy Week," which yielded £543; and they hoped to make it a permanent institution.

The meeting terminated with an enthusiastic vote of thanks to the Lady Grand President.

